# Is resilience a protective factor against the effects of the COVID-19 pandemic on mental health? Results from a national multicentric study

**DOI:** 10.1192/j.eurpsy.2021.48

**Published:** 2021-08-13

**Authors:** A. Fiorillo

**Affiliations:** Department Of Psychiatry, University of Campania “L. Vanvitelli”, Naples, Italy

**Keywords:** resilience, trauma, mental health

## Abstract

The COVID-19 pandemic is impacting on the mental health of the general population and its consequences will be long lasting. As already noted in previous epidemics, different factors can moderate the detrimental impact of a traumatogenic event on mental health. In particular, it has been found that people using problem-solving coping strategies, with an adequate social network and supported by family members, have good long-term outcomes and are able to adjust to the detrimental impact of the traumatic event. The COvid Mental hEalth Trial (COMET) network, including ten university Italian sites and the National Institute of Health, has promoted a national online survey in order to evaluate the impact of COVID-19 pandemic on the mental health of the Italian general population. In particular, the use of Internet and social media, the duration of the exposure to COVID-19 related containment measures, the different levels of post-traumatic growth and the variety of coping strategies adopted have been considered as possible mediators of the resilience styles adopted. In our sample, participants from the general population reported a good level of resilience compared with people with pre-existing mental or physical disorders. This data should be taken seriously in consideration in order to develop appropriate psychosocial interventions for supporting resilience in people at high-risk in order to mitigate the detrimental impact of the pandemic.

**Disclosure:**

No significant relationships.

